# Retraction Note: LncRNA LINC00520 aggravates cell proliferation and migration in lung adenocarcinoma via a positive feedback loop

**DOI:** 10.1186/s12890-024-03158-8

**Published:** 2024-07-16

**Authors:** Wen Huang, Xinxing Wang, Fubing Wu, Fanggui Xu

**Affiliations:** 1 Department of Oncology, The Fourth Affiliated Hospital of Nanjing Medical University, Nanjing, 210000 Jiangsu China; 2 Department of Oncology, Sir Run Run Hospital of Nanjing Medical University, Jiangning District, 109 Longmian Avenue, Nanjing, China

**Retraction Note: BMC Pulm Med 21, 287 (2021)**

**https://doi.org/10.1186/s12890-021-01657-6**

The Editor has retracted this article. After publication, the authors contacted the journal stating that the colony formation (Figs. 1F and 4C) and wound healing (Figs. 1J and 4H) assay images were incorrect, and explained that the in vitro experiments were outsourced to a third party.

The Editor therefore no longer has confidence in the presented data.

None of the authors have responded to the Editor about this retraction notice.

